# The link between soda intake and asthma: science points to the high-fructose corn syrup, not the preservatives: a commentary

**DOI:** 10.1038/nutd.2016.46

**Published:** 2016-11-28

**Authors:** L R DeChristopher, J Uribarri, K L Tucker

**Affiliations:** 1 NY Medical College, Valhalla, NY, USA; 2 Department of Medicine, the Icahn School of Medicine at Mount Sinai, New York, USA; 3 Department of Clinical Laboratory and Nutritional Sciences, University of Massachusetts, Lowell, MA, USA

## Abstract

Recent research conducted by investigators at the National Center for Chronic Disease Prevention and Health Promotion—a division of the US Centers for Disease Control and Prevention (CDC)—found that 'Regular-Soda Intake, Independent of Weight Status, is Associated with Asthma among US High School Students.' On the basis of their review of prior studies, researchers hypothesized that the association may be due to high intake of sodium benzoate, a commonly used preservative in US soft drinks. But a closer look at these prior research studies suggests that there is no strong scientific evidence that the preservatives in US soft drinks are associated with asthma. Importantly, other recent research suggests that the association may be with the unpaired (excess free) fructose in high fructose corn syrup.

## Introduction

In 2013, researchers at the National Center for Chronic Disease Prevention and Health Promotion – a division of the US Centers for Disease Control and Prevention (CDC) – found that 'Regular-Soda Intake, Independent of Weight Status, is Associated with Asthma among US High School Students.' The study was based on data collected from 15 960 students (grades 9 through 12) who participated in the 2009 National Youth Risk Behavior Survey. Results showed that students who drank regular soda three or more times per day were 64% more likely to have asthma relative to never consumers, and independent of potential confounders, including smoking and weight status; twice per day drinkers were 28% more likely to have asthma than never consumers.^1^ Further, asthma prevalence was 14.7% among students who drank regular soda three or more times per day versus 9.7% among never consumers.^1^

The biochemical mechanisms that underlie this association remain unclear, as research to better understand how soft drink consumption can adversely affect respiratory health has been limited. In the above referenced study, researchers suggested that 'one possible reason for finding the significant association between soda intake and current asthma might be the presence of food preservatives in sodas and other foods, such as sodium benzoate or sulphites (also sulfites), as most sodas contain sodium benzoate as a preservative, which has been associated with negative reactions and worsening of asthma symptoms.'^1^ CDC researchers developed this hypothesis based on their review of prior research.

### A commentary

However, a closer look at these prior studies indicates that there is no strong evidence that preservatives in US soft drinks cause asthma. For example, in the 1980 study titled, 'Sulphur dioxide in foods and beverages: Its use as a preservative and its effect on asthma,' Freedman et. al. analyzed asthma associations with sulfur dioxide.^1, 2^ However, sulfur dioxide is not relevant to association studies with US soft drinks, as sulfur dioxide is not used in US soft drinks.^3, 4^ Its use is primarily as a preservative in dried fruits.^5^ Notably, the most widely used preservatives in US soft drinks are sodium benzoate and potassium benzoate.^3, 4^ The only thing these molecules have in common is that they are all preservatives.^6, 7^ Therefore, this reference does not support an assertion that 'sodium benzoate has been associated with negative reactions and worsening asthma symptoms.'^1^ This same limitation also exists for another referenced study conducted in 1993 by Steinman *et al.* titled, 'Sulphur dioxide sensitivity in South African asthmatic children.'^1, 8^

A closer look at a 1985 study by Genton *et al.*^9^—which included oral provocation testing with sodium benzoate—provides meaningful insights. These researchers conducted oral provocation tests with 34 patients (17 with asthma and 17 with urticaria). Of the 17 patients with asthma, one had a positive result to the sodium benzoate oral provocation test (OPT).^9^ This very small sample size and single positive OPT result with sodium benzoate limits the reliability, and generalizability of this study, particularly as no other study, that we know of, has replicated this finding. Therefore, it is possible that the single positive OPT may have been a false positive.

A review of the 1985 study by Steinman *et al.*^10^ titled, 'The effects of soft-drink preservatives on asthmatic children,' conducted with children in Cape Town South Africa is also relevant. Specifically, 'parents of asthmatic children were asked to complete a questionnaire, which listed the following: soft drink usually ingested; cough and/or wheeze following ingestion; development of a rash following ingestion and type of rash; time elapsed before onset of symptoms; the duration of symptoms; and who observed the reactions.'^10^ They reported that many children were sensitive to sulphites and some to sodium benzoate, but parental report among symptomatic children could be subject to recall bias. According to the latest 'Guidelines for the Diagnosis and Management of Food Allergy in the US, published by the National Institute of Allergy and Infectious Diseases (NIAID)—a division of the National Institutes of Health (NIH), an oral food challenge, alongside skin prick tests and measurement of allergen specific IgE in the serum, is the gold standard to identify and diagnose a food allergy. Food elimination experiments may also identify sensitivities.^11^ In the study by Steinman *et al.*, no oral provocations, skin prick tests, IgE testing or food elimination experiments were used to confirm parents' responses.^12^

The findings of another cross-sectional study (2005) with 1321 New Zealand children, aged 10–12 years, may provide important insights. This study, which is part of the International Study of Asthma and Allergies in Childhood known as ISAAC, sought to test the hypothesis that the consumption of fast food is related to asthma prevalence. After adjusting for potential lifestyle confounders, researchers found no association between 'fizzy': drinks and ever having asthma.^13^ The finding that 'fizzy' drink consumption in New Zealand was not associated with asthma supports the possibility that another ingredient—other than preservatives—is responsible for the difference in results between the New Zealand ISAAC study and the US CDC study. This may be due to the use of different sweeteners between these two countries. In New Zealand, 'fizzy' drinks are sweetened with cane sugar (sucrose),^14^ whereas in the US, non-diet soft drinks are sweetened with high fructose corn syrup (HFCS).^15^ Therefore, it is possible that the HFCS in US soft drinks is responsible for the increased asthma prevalence among regular consumers of non-diet soft drinks.

Notably, in a recent longitudinal birth cohort study (*n*=60 466) in Denmark, artificially sweetened soft drink beverages (ASB) were associated with asthma, but sugar-sweetened beverages (SSB) were not. Consumption of carbonated ASB, but not SSB, by mothers during pregnancy, was associated with registry-based asthma and self-reported allergic rhinitis outcomes in children at age 7, while early childhood (18 months) outcomes were related to non-carbonated ASB. However, mothers who drank ASB were also significantly more likely to smoke during pregnancy, and it is possible that they were more likely to smoke indoors, while at home. Therefore, in-home smoke may have confounded their results.^16^ Importantly, this study provides further evidence that preservatives in soft drinks are not associated with asthma, as both ASB and SSB contain preservatives.

Another scientific method recommended by the NIAID which can be used to reliably assess food sensitivity is a food elimination diet.^11^ In the US, the results of a rigorous food elimination diet provided evidence that HFCS is associated with chronic airway mucus hypersecretion, chronic bronchitis and asthma.^17^ This led to development of the 'fructositis' hypothesis which raises the possibility that consumption of unpaired (excess free) fructose, as occurs in high fructose corn syrup and certain other foods, contributes to the intestinal *in situ* formation of pro-inflammatory advanced glycation end-products (AGE) due to underlying fructose malabsorption.^17, 18, 19, 20^ Other sources of unpaired (excess free) fructose include apple juice, fruit drinks, apples, pears, watermelons, mangoes,^21^ and agave syrup.^22^ Fruit drinks are included here because many varieties are sweetened with HFCS and contain apple juice as a main ingredient. Notably, apple juice contains a 2:1 ratio of fructose to glucose.^21^ Importantly, individuals who are fructose malabsorbers cannot adequately absorb fructose that is unaccompanied by an equal amount of glucose.^23, 24, 25, 26^

With high levels of unabsorbed fructose and favorable alkaline conditions in the upper intestine, the unabsorbed fructose glycates partially digested dietary proteins, and turns them into antigens (enFruAGE), which are capable of pro-inflammatory signaling that can trigger airway mucus hypersecretion, and promote chronic respiratory conditions and other comorbidities. It is possible that the intestinal *in situ* formation of enFruAGE by the non-enzymatic Maillard reaction contributes to higher concentrations of pro-inflammatory advanced glycation end-products that are more insidious than dietary AGE (dAGE) or AGE formed in the systemic circulation under high glucose conditions. Unlike conditions present during food processing and in the systemic circulation, proteins in the digestive tract are at various levels of digestion. Therefore, they have a greater number of exposed free amino groups, which are capable of reacting with the carbonyls of unabsorbed fructose.^17, 18, 19, 20^

The intestinal *in situ* formation of enFruAGE may produce AGE that are smaller and more capable of diffusing out of the vascular compartment and into surrounding tissues than AGE in the diet or AGE formed in the systemic circulation. Once there, enFruAGE could initiate an inflammatory cascade, which promotes tissue damage. Because they have the potential of being smaller and more concentrated, enFruAGE could more readily contribute to plaque formation and cardiovascular disease, than AGE formed in the systemic circulation. Because of their small size, enFruAGE may infiltrate joint tissue resulting in synovitis, arthritis and joint pain. They may accumulate in the pancreas resulting in pancreatitis, insulin insufficiency and type 2 diabetes (T2D). EnFruAGE may be harbingers of chronic inflammation.^17, 18, 19, 20^

Further, certain transition metals are known to accelerate the Maillard reaction. For example, iron (Fe2+) from iron rich foods will speed up the Maillard reaction.^27^ Importantly, anionic ligands, including phosphates and bicarbonate, are potent catalysts of glycation at specific sites on proteins.^28, 29, 30^ Some soft drinks, particularly colas, contain phosphoric acid. Therefore, phosphates from soda could serve as catalysts of the Maillard reaction in the duodenum and jejunum. Additionally, bicarbonate injected into the duodenum from the pancreas after a meal, to neutralize the acidic bolus from the stomach, could catalyze the Maillard reaction.^28, 29, 30^ It may be that malate from malic acid in apple juice has similar effects. These are relevant and important kinetic parameters, as they can have hastening effects on the rate limiting steps of glycation,^28, 29, 30^ and may precipitate the intestinal *in situ* formation of enFruAGE within the timeframe of digestion.^17, 18, 19, 20^ Importantly, fructose malabsorption (FM) underlies this reactivity, as FM occurs only after consumption of unpaired (excess free) fructose, but not with sucrose, or equal amounts of fructose and glucose monomers.^23, 24, 25, 26^

The greater the concentration of unabsorbed fructose remaining in the upper intestine, the greater is the potential for enFruAGE formation via the Maillard reaction. Within the past five years, independent researchers found that the fructose in US soft drinks exceeds levels that are generally recognized as safe (GRAS).^31, 32^ This may be an important finding and may be relevant to the US CDC's discovery that soft drink consumption by US high-schoolers is associated with asthma.^1^ In 2011 and again in 2014, University of California, Los Angeles (UCLA) researchers commissioned independent laboratories to measure the amount of fructose in popular US soft drinks. They consistently found that the HFCS used to sweeten popular soft drinks contains more fructose than is Food and Drug Administration-approved and GRAS.^31, 32^

These findings are alarming as up to one half of the population is unable to completely absorb a load of 25 g of unpaired (excess free) fructose^23^—a daily dosage that is easily reached at present levels of HFCS consumption, particularly when HFCS contains outsize amounts of fructose.^15, 31, 32^ Average per capita HFCS consumption has been estimated at just under 1 lb per week.^33^ However, this longstanding trend may be changing, as recent clinical trial research has linked HFCS to metabolic syndrome and T2D.^34, 35^ Increasingly popular alternatives to HFCS include agave syrup (>60% fructose)^22, 36^ and crystalline fructose.^37^ Unfortunately, this shift will have no effect on adverse outcomes associated with fructose malabsorption, as these alternatives are also sources of unpaired excess free fructose, that is, fructose that is consumed in excess of glucose.^22, 36, 37^

The 'fructositis' hypothesis was recently investigated with nationally representative data from the 2003–2006 National Health and Nutrition Examination Survey data (NHANES). The study was designed to epidemiologically test the hypothesis that consumption of unpaired excess free fructose, but not sucrose, is associated with asthma.^18^ Results support the proposed hypothesis. This cross-sectional study of 1961 children, aged 2–9 years, showed that children who regularly consumed (⩾5 times per week) any combination of high fructose corn syrup sweetened soft drinks, fruit drinks and apple juice were five times more likely to have asthma relative to seldom/ never consumers, independent of potential confounders including atopy. There was no association with orange juice.^18^

In addition to the asthma investigation with children, we found that adults, ages 20–55 years (*n*=2801), who regularly consumed HFCS sweetened soft drinks, were nearly twice as likely to have chronic bronchitis than never/seldom consumers.^19^ Further, young adults, ages 20–30 years (*n*=2801), who regularly consumed any combination of HFCS sweetened soft drinks, fruit drinks and apple juice were three times more likely to suffer from non-wear and tear, non-age associated autoimmune arthritis than never/seldom consumers.^20^ Again, we found that orange juice, which contains sucrose and a relatively equal ratio of fructose to glucose, was not associated with the outcomes investigated,^18, 19, 20^ nor were diet soft drinks or diet fruit drinks.^19, 20^

These are not the only studies to link apple juice, but not orange juice, with adverse respiratory conditions. A study of 1111 mother-child pairs from Project Viva—a longitudinal pre-birth cohort—found that (at 2 years of age) child intake of juice, excluding orange juice increased asthma risk by 34%.^38^ In another study with children in the Netherlands, high consumption of 100% fruit juice (type not distinguished) was associated with increased risk of asthma.^39^ Therefore, it is possible that the association was driven by apple juice, rather than other 100% fruit juices.^40^ Results from each of these studies, when considered together, provide support for the hypothesis that the association between US soft drinks and asthma is due to the unpaired excess free fructose in HFCS, rather than preservatives.

In the aforementioned ISAAC study with 'fizzy' drinks, another of the study's findings with fast food, and in particular hamburgers, provides evidence that dietary AGE - and by extension all AGE capable of pro-inflammatory signaling — may play a role in asthma, as children who consumed hamburgers once or more per week were nearly twice as likely to have asthma symptoms or a history of asthma symptoms as never consumers.^13^ This hypothesis is supported by the fact that grilled meats have relatively high levels of dAGE.^41^

Interestingly, results from a nested case-control study conducted within the European Prospective Investigation of Cancer in Norfolk (EPIC-Norfolk) - showed that participants with the highest consumption level of meat and meat products had twice the risk for inflammatory polyarthritis as seldom/ never consumers, after adjusting for potential confounders.^42^ Notably, arthritis is a common comorbidity of asthma. In a cross-sectional study (*n*=22 172) with nationally representative survey data (NHANES) between 2003 and 2010, thirty one percent of asthma sufferers (10%) had arthritis.^43^ The population attributable risk of chronic comorbidities, including asthma, arthritis, metabolic syndrome, and T2D, due to regular consumption of high fructose corn syrup sweetened soft drinks, and other high excess free fructose foods and beverages remains unknown. Interestingly, research by the Mayo Clinic found that children and adults with asthma were significantly more likely to be diagnosed with T2D, and adults with asthma were significantly more likely to be diagnosed with coronary heart disease.^44^

As this commentary was being written, the US CDC reported results of another epidemiologic study of sugar sweetened beverages and asthma. They analyzed 2013 Behavioral Risk Factor Surveillance data for 146 990 adults (⩾18 years) from 23 states and the District of Columbia. SSB was defined as soda, fruit drink, sweet tea, and sports/energy drinks. They found that non-obese adults who consumed SSB⩾2 times/day were 66% more likely to have asthma as non-consumers—independent of lifestyle factors.^45^

In summary, this commentary is a call for clinical and biochemical research that aims to confirm and clarify the mechanisms responsible for the association between regular intake of high fructose corn syrup sweetened soft drinks and chronic respiratory conditions and other comorbidities. Existing evidence does not support the hypothesis that the preservatives in soda are responsible for the increased asthma prevalence among regular consumers of non-diet soft drinks. The greater preponderance of scientific evidence supports the hypothesis that the association is with the high fructose corn syrup, possibly due to underlying fructose malabsorption and the intestinal *in situ* formation of advanced glycation end-products, due to the higher fructose to glucose ratio than is seen with sucrose. Further, recent murine based research provides evidence of AGE formation in the jejunum.^46, 47^ The recommendation that children and adolescents limit their intake of soft drinks would be a fine strategy to minimize asthma risks if the problem was with high concentrations of sodium/potassium benzoate. However, scientific evidence is pointing us in another direction. Importantly, in addition to high fructose corn syrup sweetened soft drinks, this includes apple juice, a widely used '100% fruit juice.' Research into the greater consequences of fructose malabsorption, beyond increased gas and bloating are long overdue.
